# Anuran genome size evolution is driven by relatively recent retrotransposon activity and by life history

**DOI:** 10.1186/s12864-025-12414-y

**Published:** 2025-12-18

**Authors:** Andrew O. Rubio, John H. Neddermeyer, Aakash James, Nicholas Janz, Jonathan A. Rader, Marc Tollis, Adam M. M. Stuckert

**Affiliations:** 1 Department of Biology and Biochemistry, University of Houston, Houston, TX 77204 USA; 2 School of Informatics, Computing, and Cyber Systems, Northern Arizona University, Flagstaff, AZ 86011 USA; 3 Department of Biology and Chemistry, Texas A&M International University, Laredo, TX 78041 USA

**Keywords:** Genome evolution, Amphibians, Repeat elements, Retroelements, DNA transposons, Repeat landscape, Transposable elements, Ty3

## Abstract

**Background:**

Transposable elements (TEs), often referred to as ‘selfish genetic elements’, hijack their host’s genetic machinery to replicate themselves within the host genome, and are primary contributors to genome size in vertebrates. In particular, frogs and toads (Order: Anura) are well known for not only having large genomes, but for having genomes with drastic variation in size (e.g., *Scaphiopus couchii*: 0.48 Gbp, *Rana muscosa* 10 Gbp). This discrepancy in size is due in part to relative differences in the proliferation and success of TEs across anuran genomes. In this study, we ask: do specific TE families drive genome size variation and are these patterns phylogenetically-constrained? To answer this, we use 61 publicly available anuran reference genome assemblies, comprising 22 anuran families, to investigate the effects of TEs on the evolution of anuran genome size. In addition, we used repeat landscapes to analyze TE activity in the context of anuran evolutionary history, in order to understand how lineage-specific TE activity affects genome size variation in anurans. Finally, we examined how life history traits that have been predicted to be constrained by genome size, larval period and clutch size, are associated with TEs.

**Results:**

Our results suggest that copy number of elements and total content of several TEs families (L1/CIN4, Ty1/Copia, Ty3/DIRS1, hobo-Activator) are associated with increased genome size across anurans. We further found that the copy numbers of recently active Ty3 retrotransposons are correlated with increased genome size, suggesting that expansions of this family of TE have had a distinct effect on the size of the anuran genome across evolutionary time. Finally, we found interactions between several TE families (hobo-Activator, PiggyBac, L2/CR1/Rex, and SINEs) and larval period, indicating that developmental time may be a major constraint on genome size and species with additional constraints may more effectively purge TEs.

**Conclusion:**

Our findings underscore the pivotal roles of both the activity of specific TE families (L1/CIN4, Ty1/Copia, Ty3/DIRS1, hobo-Activator) and developmental constraints in amphibian genome size evolution.

**Supplementary Information:**

The online version contains supplementary material available at 10.1186/s12864-025-12414-y.

## Background

Genomes contain all the genetic material that organisms use during development and throughout life. However, in many taxa the protein-coding regions of the genome make up a small proportion of the genomic material [1]. There is also drastic variation in genome size across taxa. For example, genome sizes in vertebrates range from several hundred Mbp to over 100 Gbp [2, 3]. Potential costs to increased genome size may include enlarged cell nuclei [4, 5], slower cell replication [6], changes in organ structures such as the liver and heart which can lead to decreased function and efficiency [7], and an overall larger number of deleterious mutational targets [8, 9].

Increased genome size in eukaryotes is often attributed to the proliferation of interspersed ‘selfish’ repetitive DNA sequences known as transposable elements (TEs) [10, 11]. TEs hijack the host genetic machinery to replicate within various parts of the host genome [12], potentially leading to mutations that cause gene disruption or regulatory changes. TE-caused mutations are more likely to be detrimental than beneficial [13–16], and include structural changes like deletions, duplications, and inversions [17]. Consequently, organisms have evolved a suite of mechanisms to protect the integrity of their genomes by purging or inactivating TEs, including RNA interference (RNAi), DNA methylation, histone modifications, and PIWI-interacting RNAs (piRNAs) [18–20].

Duplication events, either chromosomal or whole genome, may also be a driver of genome expansion. Duplication events not only directly increase DNA content but may also trigger TE bursts by disrupting epigenetic regulation or genomic stability [21, 22]. For instance, in Corydoradinae catfishes, both a whole genome duplication and TE proliferation have been shown to contribute significantly to genome size increases [23]. Similarly, in Atlantic salmon, a recent whole-genome duplication was followed by rediploidization and a burst of TE activity, suggesting that genome duplications can create conditions that promote TE mobilization [24]. These examples highlight how both WGD and TE activity can drive genome expansion. We note, however, that duplication events are a common source of genomic content in some lineages (e.g., plants), while very uncommon in other lineages (e.g., amphibians).

Transposable elements are diverse and can be categorized into two major classes: retroelements which include Short Interspersed Nuclear Elements (SINEs), Long Interspersed Nuclear Elements (LINEs), and Long Terminal Repeat retrotransposons (LTR elements), and DNA transposons which include PiggyBac, hobo-Activator, Tourist/Harbinger elements. Retroelements move within the genome by using a cut-and-paste mechanism that allows repetitive elements to first be transcribed into RNA, then reverse-transcribed into DNA, and finally integrated back into the genome [25]. DNA transposons use a copy-and-paste mechanism that allows elements to directly be removed from one location and inserted to another [25]. Both classes of TEs can become integrated into a host genome and then proliferate before being silenced or purged by host protective machinery. As a result, bursts of TE activity are predicted to inflate genome size [26]. Substantial effort has been devoted to investigating the evolutionary dynamics of TEs across various taxonomic groups [27–31] as elucidating TE abundance, activity, and diversity in a phylogenetic context can allow us to trace TE evolutionary history and understand their impact on genome architecture [18, 32, 33].

Within vertebrates, amphibians in particular have evolved both increased genome sizes as well as dramatic variation in genome size, ranging from 0.48 Gbp [34] to over 110 Gbp [35] in length. This variation in genome size is due in part to the relative abundances of TEs [3, 36]. Conversely, genome contraction in amphibians can occur through purging genomic content and a loss or suppression of TEs [37]. Evidence suggests a role for recombination in this purging process, as genomic regions with higher recombination rates tend to exhibit lower TE densities [38, 39]. In Amphibians, genome size evolution appears to follow distinct patterns across the three major lineages; frogs and toads (Order Anura) and caecilians (Order Gymnophiona) exhibit gradual changes consistent with Brownian motion, while salamanders (Order Urodela) experienced an early, pronounced expansion resulting in uniquely large genomes that do not overlap with those of other amphibians [40]. Evidence suggests that the drastic increase in Urodela genome size is primarily driven by LTR elements, such as Ty3 and ERV [41–43].

Within the order Anura, genome assemblies of early-diverging lineages of appear to contain lower proportions of TEs (e.g., 34.5% of the *Xenopus tropicalis* genome; 48% of the *Nanorana parkeri* genome), whereas genomes of more recently diverged anuran lineages consist of high proportions of TEs (e.g., 77% of the *Ranitomeya imitator* genome; approximately 70% of the *Oophaga pumilio* genome) [44–47]. It is worth noting that both of these *R. imitator* and *O. pumilio* are members of the family Dendrobatidae which shows dramatic genome size diversity [48]. Interestingly, certain classes of TEs such as Ty3 (more commonly known by its problematic and ethnically denigrating name Gypsy, see a discussion of this in [49]) are similarly abundant in both the *R. imitator* and *O. pumilio* genomes (1.2 Gbp and 1 Gbp, respectively), but not in genomes from other families, such as ranids [50] whereas the abundance of other classes of TEs differs dramatically between these genomes, such as the hAt DNA transposons (1.1 Gbp and 255 Mbp, respectively). In other systems, bursts of specific families of TEs, such as LTR retrotransposons, have driven increases in genome size, and thus [32, 51–54], the fundamental question is whether genome size is driven by specific TE families within amphibians.

Given the physiological and mutational effects of genome size mentioned above, changes in genome size and TE content may also be linked to life history traits such as development and ecological adaptations in amphibians. For example, many salamanders with larger genomes are exclusively associated with permanent aquatic habitats or breed terrestrially, possibly because environmental stability allows for slower development [55, 56]. Another study found that genome size of members of the Dendrobatidae family is positively correlated with snout-vent-length, oocyte number, and clutch size [48]. These characteristics highlight the dynamic role transposable elements are hypothesized to play in shaping not just genome size but also physiological, developmental, and metabolic traits across amphibians. The dramatic variation in genome size within anurans makes this clade a promising model to study the evolution of TEs and may shed light on key biological questions regarding the origins of as well as the constraints on genome size.

**Fig. 1:**
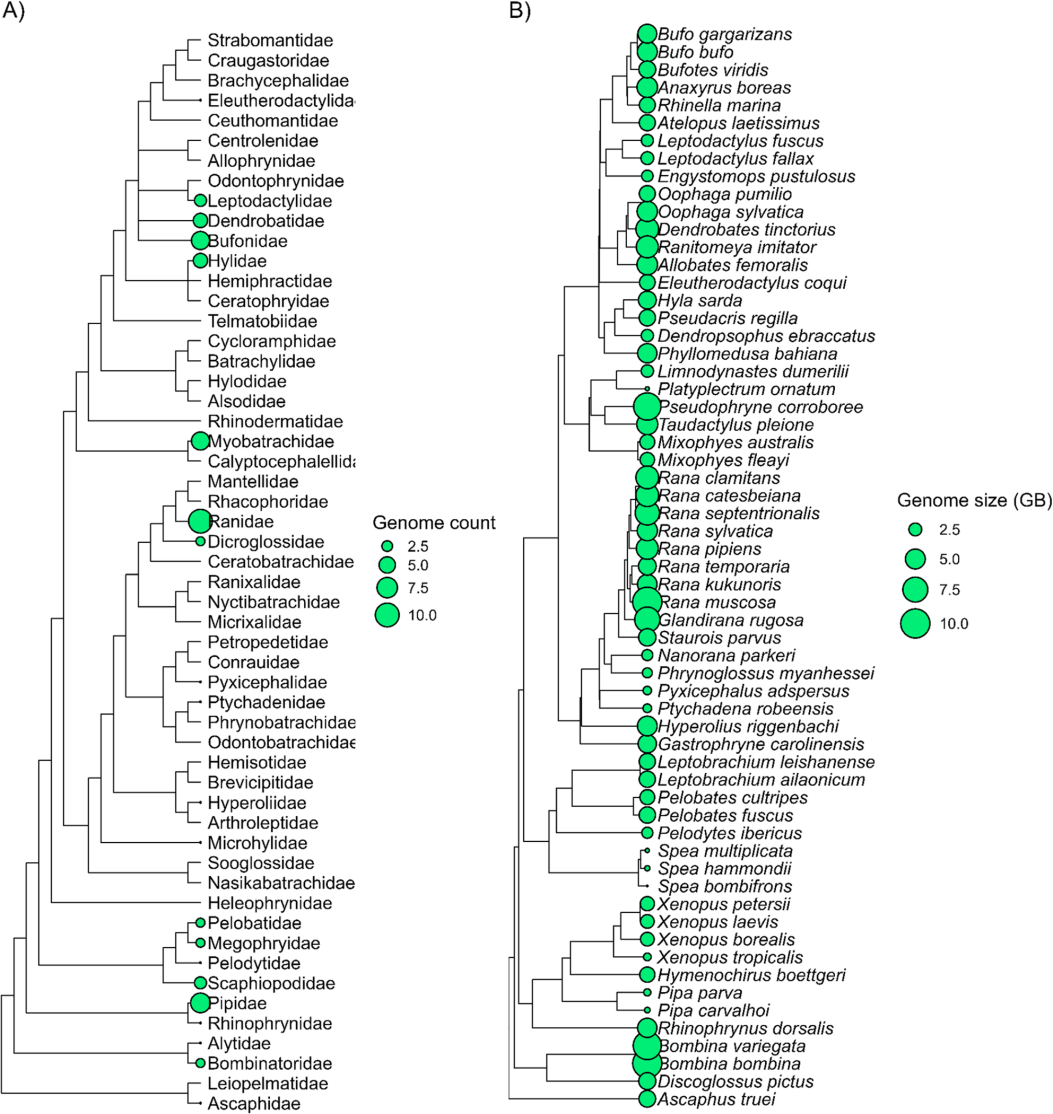
**(A)** Number of published genomes in NCBI by anuran family as of October 29th, 2024. **(B)** Genome size for each species included in the analysis. More detail on genome assemblies (methodology, N50 stats, BUSCO scores, etc.) can be found in Table 1

Here, we use 61 publicly available anuran reference genome assemblies, comprising 22 families (Fig. 1A and B), to take a broad phylogenetic approach to understand why amphibian genomes are so variable in size. We hypothesized that the proliferation of TEs within certain lineages has been a driving factor in genome size variation in anurans. Although genomic duplication events and polyploidy may contribute to genome size expansion, both of these appear to be rare in anurans [57, 58], and largely absent in available anuran genomes. We predicted that species with larger genomes will have higher copy numbers and greater overall proportion of TEs in the genome, particularly long elements such as LTR retrotransposons. Understanding the abundance, activity, and diversity of TEs will allow us to determine if specific families of TEs are driving variable genome size in anurans. We additionally test if clutch size or larval period are associated with TE content, as we predict both traits may be constrained by genome size. Our results suggest that several TE families are associated with increased genome size in anurans, four of which are in the retroelement class (Penelope, L1/CIN4, Ty1/Copia, and Ty3/DIRS) and one of which is in DNA transposon class (hobo-Activator). Finally, we found that across anuran genomes the copy numbers of recently active Ty3 retrotransposons are significantly positively correlated with genome size, suggesting that expansions of this family of TE have had a distinct effect on the size of the anuran genome across evolutionary time.

## Methods

### De novo repeat annotation of anuran genome assemblies

We used the NCBI datasets command-line tools [59] to download reference genome assemblies using the command “datasets download genome taxon anura --reference --include genome” (last accessed October 29th, 2024). This yielded 64 genome assemblies for 64 species. We examined genome size and contig N50 and removed assemblies with contig N50 < 1k (*Pseudis tocantins*: GCA_023970735.1, *Scaphiopus couchii*: GCA_009364435.1, and *Scaphiopus holbrookii*: GCA_009364455.1) leaving us with 61 assemblies (Table 1; Fig. 1A and B). We used the program RepeatModeler2 version 2.0.5 [60] to model TEs in each genome assembly by building species-specific repeat libraries with the search engine option “ncbi”. We used the RFSB classifier from the program TransposonUltimate [61] to identify transposon elements that could not be classified and were labeled by RepeatModeler2 as “Unknown”. Newly identified TEs were then programmatically added to the consensus output from RepeatModeler2. In addition, we extracted known vertebrate repetitive elements from Dfam version 1.0.5 [62] and merged this with the classified consensus output from RepeatModeler2 via concatenation. We then queried each reference genome against its species-specific repeat library using the program RepeatMasker version 4.1.7 [63] with the following flags,“-xsmall” which softmasks genomes by denoting repeat regions in lower case and non-repetitive regions in capitals, “-no_is” which skips bacterial insertion element check, “-a” which shows the alignments in an alignment output file, and “q” which implements a quick search. Output provided by RepeatMasker reflects superfamily-level classifications, which may group distinct families under a shared label. Throughout the manuscript, we refer to the number of RepeatMasker hits as the number of insertions.

**Table 1:** Anuran reference genome assemblies from the National center for biotechnology information (NCBI). BUSCO scores were calculated using compleasm version 0.2.7 and the tetrapoda_odb12 database

Family	Species	Assembly Accession	Genome Size (Gbp)	Assembly Stats Contig N50 (Kbp)	BUSCO Score	Assembly Sequencing Tech		
						Short read	Long read	3D structure
Alytidae	*Discoglossus pictus*	GCA_033576555.1	3.87	3,043.6	86.61%,2.61%,2.47%,0.04%,8.27%	X	X	X
Aromobatidae	*Allobates femoralis*	GCF_028390025.1	5.32	879.1	93.74%,0.85%,0.73%,0.00%,4.68%	X	X	
Ascaphidae	*Ascaphus truei*	GCA_037306005.1	3.72	981.5	77.41%,0.43%,7.40%,0.05%,14.71%		X	X
Bombinatoridae	*Bombina bombina*	GCA_029206835.1	10.02	3,759.9	87.36%,1.90%,3.06%,0.02%,7.66%		X	
	*Bombina variegata*	GCA_029574335.1	9.37	1,741.7	94.33%,0.73%,0.60%,0.00%,4.34%		X	X
Bufonidae	*Bufo gargarizans*	GCA_011038615.1	4.55	1,738.3	83.18%,0.84%,6.58%,0.00%,9.41%		X	
	*Bufo bufo*	GCA_019447015.1	5.04	3,959.8	88.30%,1.97%,3.11%,0.00%,6.62%			
	*Bufotes viridis*	GCA_037952925.1	3.8	1,872.4	87.62%,2.01%,3.52%,0.05%,6.79%	X	X	
	*Rhinella marina*	GCA_039654945.1	3.47	860.7	42.09%,54.12%,0.44%,0.00%,3.34%		X	X
	*Anaxyrus boreas*	GCA_042186555.1	5.28	2,844.7	93.08%,1.05%,1.03%,0.00%,4.84%	X	X	X
	*Atelopus laetissimus*	GCF_000935625.1	3.51	31,028.3	89.88%,0.68%,3.22%,0.00%,6.22%		X	
Dendrobatidae	*Ranitomeya imitator*	GCA_027410445.1	5.96	13,907.6	60.71%,0.57%,16.20%,0.30%,22.21%		X	X
	*Dendrobates tinctorius*	GCA_041080635.1	6.36	32,538.6	93.14%,1.17%,0.96%,0.00%,4.73%		X	
	*Oophaga pumilio*	GCA_933207985.1	3.49	5.8	88.32%,2.58%,2.45%,0.00%,6.65%	X		
	*Oophaga sylvatica*	GCF_035609145.1	5.19	97.8	93.26%,1.17%,0.68%,0.00%,4.89%	X	X	
Dicroglossidae	*Phrynoglossus myanhessei*	GCA_038501925.1	1.83	1.5	93.94%,0.91%,0.64%,0.00%,4.52%	X		
Dicroglossidae	*Nanorana parkeri*	GCA_900303285.2	2.05	32.8	92.57%,1.62%,1.24%,0.00%,4.57%	X		
Eleutherodactylidae	*Eleutherodactylus coqui*	GCA_027789725.1	3.37	10,389.0	94.13%,1.51%,0.73%,0.00%,3.63%		X	X
Hylidae	*Hyla sarda*	GCA_033576535.1	4.14	3,809.3	91.91%,1.42%,1.65%,0.02%,5.00%		X	
	*Pseudacris regilla*	GCA_041430395.1	3.6	7,148.8	93.85%,1.30%,0.82%,0.00%,4.04%	X	X	X
	*Dendropsophus ebraccatus*	GCA_951230385.1	2.35	10,560.9	89.95%,0.48%,3.40%,0.04%,6.14%	X	X	X
	*Phyllomedusa bahiana*	GCA_964205295.1	4.74	78.5	88.80%,3.24%,2.40%,0.02%,5.55%		X	
Hyperoliidae	*Hyperolius riggenbachi*	GCA_036172795.1	4.92	2,674.8	62.80%,0.66%,10.33%,0.04%,26.18%		X	X
Leptodactylidae	*Engystomops pustulosus*	GCA_018994145.1	2.16	21,805.6	85.27%,1.33%,2.40%,0.00%,10.99%		X	X
	*Leptodactylus fuscus*	GCA_027917425.1	2.31	5,565.7	92.69%,2.63%,0.71%,0.00%,3.97%		X	X
	*Leptodactylus fallax*	GCF_027358695.1	2.51	4,630.2	94.58%,0.62%,0.55%,0.00%,4.25%		X	
Megophryidae	*Leptobrachium ailaonicum*	GCA_019650415.1	3.54	821.1	28.05%,0.05%,34.86%,0.53%,36.51%		X	
	*Leptobrachium leishanense*	GCA_036250195.1	3.55	1,946.3	94.63%,0.71%,0.71%,0.00%,3.95%		X	
Microhylidae	*Gastrophryne carolinensis*	GCA_028564925.1	4.34	4,962.6	92.34%,0.73%,1.16%,0.00%,5.78%		X	X
Myobatrachidae	*Pseudophryne corroboree*	GCA_033119425.1	8.87	6,819.1	93.40%,1.40%,0.66%,0.00%,4.53%		X	X
	*Mixophyes fleayi*	GCA_037952835.1	2.97	4,050.0	89.72%,1.67%,2.65%,0.00%,5.96%		X	X
	*Mixophyes australis*	GCF_014858855.1	3.13	10,748.0	87.27%,4.21%,1.46%,0.00%,7.06%	X	X	
	*Taudactylus pleione*	GCF_017654675.1	5.51	8,853.5	41.12%,55.10%,0.48%,0.00%,3.31%		X	
	*Limnodynastes dumerilii*	GCF_040937935.1	2.38	10.6	90.97%,1.81%,1.16%,0.02%,6.05%	X		
	*Platyplectrum ornatum*	GCF_905171775.1	1.07	4.7	91.87%,1.40%,1.08%,0.00%,5.64%	X	X	
Pelobatidae	*Pelobates fuscus*	GCA_024363595.1	3.61	29,412.6	0.00%,0.00%,0.27%,0.00%,99.73%		X	X
	*Pelobates cultripes*	GCA_031769625.1	3.09	130.4	88.69%,3.43%,1.60%,0.02%,6.26%			
Pelodytidae	*Pelodytes ibericus*	GCA_031893025.1	2.08	19,137.4	93.37%,1.26%,0.89%,0.00%,4.48%		X	X
Pipidae	*Xenopus laevis*	GCA_004786255.1	2.74	22,451.5	90.20%,0.57%,2.60%,0.00%,6.63%		X	
	*Xenopus tropicalis*	GCA_009364415.1	1.45	14,634.3	72.49%,7.36%,10.39%,0.02%,9.75%	X	X	
	*Xenopus borealis*	GCA_016617825.1	2.75	21.2	46.22%,0.32%,22.87%,0.18%,30.41%	X		
	*Hymenochirus boettgeri*	GCA_021901965.1	3.21	783.8	12.02%,0.02%,26.57%,0.53%,60.86%	X	X	
	*Xenopus petersii*	GCA_032444005.1	2.88	15,295.0	93.88%,1.08%,0.78%,0.00%,4.25%		X	X
	*Pipa carvalhoi*	GCA_043774495.1	1.19	2.0	90.97%,1.48%,1.42%,0.00%,6.14%	X		
	*Pipa parva*	GCF_905171765.1	1.37	6.2	93.70%,1.12%,0.75%,0.00%,4.43%	X		
Ptychadenidae	*Ptychadena robeensis*	GCA_038048845.1	1.59	12.4	86.50%,5.48%,2.31%,0.00%,5.71%	X		
Pyxicephalidae	*Pyxicephalus adspersus*	GCA_022657655.1	1.56	30.4	8.34%,0.09%,24.33%,0.55%,66.69%	X		
Ranidae	*Aquarana catesbeiana*	GCA_009667805.1	6.38	3,396.5	91.41%,1.87%,1.16%,0.00%,5.57%		X	
	*Rana temporaria*	GCA_009801035.1	4.11	6,264.8	71.23%,0.60%,14.60%,0.05%,13.52%			
	*Rana sylvaticus*	GCA_029215755.1	5.15	2,498.7	84.94%,8.75%,1.16%,0.00%,5.16%		X	X
	*Rana kukunoris*	GCA_036250615.1	4.83	1,704.3	94.42%,1.39%,0.52%,0.00%,3.68%	X	X	
	*Rana muscosa*	GCA_037367395.1	10.16	4,644.3	94.49%,0.59%,1.08%,0.00%,3.84%		X	
	*Rana clamitans*	GCA_040206685.1	6.36	4,403.2	93.22%,1.19%,1.05%,0.00%,4.53%		X	
	*Rana pipiens*	GCA_040894005.1	5.9	5,395.2	91.84%,1.01%,1.58%,0.00%,5.57%		X	
	*Rana septentrionalis*	GCF_027579735.1	7.1	3,139.3	89.63%,3.34%,2.29%,0.00%,4.73%		X	
	*Staurois parvus*	GCF_029499605.1	3.98	611.2	92.17%,0.87%,1.44%,0.00%,5.51%		X	
	*Glandirana rugosa*	GCF_036172605.1	7.63	11.0	93.85%,1.28%,0.69%,0.00%,4.18%	X	X	
Rhinophrynidae	*Rhinophrynus dorsalis*	GCA_018402905.1	4.75	12,994.9	83.03%,1.24%,6.14%,0.05%,9.53%		X	X
Scaphiopodidae	*Spea bombifrons*	GCA_025379985.1	0.99	21,170.4	58.71%,29.52%,4.77%,0.00%,7.01%		X	X
	*Spea hammondii*	GCA_947044405.1	1.16	14,335.8	94.31%,0.69%,1.07%,0.00%,3.93%	X	X	X
	*Spea multiplicata*	GCF_000004195.4	1.08	30.7	95.71%,0.73%,0.64%,0.00%,2.92%		X	

#### Correlates of genome size, transposable elements, GC region, and life history and ecological traits

We used Phylogenetic Generalized Least Squares (PGLS) to control for phylogenetic relatedness. We ran three separate models to test 1) whether specific families of TE are associated with genome size, 2) whether total GC content is associated with genome size and total of TEs, and 3) whether specific families of TE are associated with life history and ecological traits. We derived a phylogenetic tree of the species included in this study by using the ‘drop.tip’ function from the R package “ape” [64] to extract only the species in our study from a species-level frog tree based on hundreds of loci [65]. We then employed the “corPagel” function from “ape” to infer phylogenetic correlation structure, setting the fixed parameter to FALSE to estimate the lambda (λ) parameter from the data. This approach allowed our models to determine the optimal estimate of how much of the phylogenetic signal influences genome size. Finally, we fit PGLS models using the “nlme” R package [66], specifying the correlation argument to account for the phylogenetic correlation structure. We controlled for contig N50 for each genome assembly to isolate confounding factors by including it as a predictor variable in our models. For all three models, we used a Benjamini-Hochberg (BH) procedure [67] to adjust the *p*-value and control for False Discovery Rate (FDR) due to multiple comparisons, p.adjusted.method = “BH” function in base R.

We used a PGLS to test whether specific families of TE are associated with genome size. To do this we extracted the number of elements for each TE class, length occupied by each TE class, and proportion of TE relative to the entire genome from the RepeatMasker summary table output (Table S1). We included all classes of TEs provided by RepeatMasker as predictor variables in each of the three models. To estimate the proportion of genome size variation explained by individual TE families, we calculated pseudo-*R*^2^ values for each univariate PGLS model. Pseudo-*R*^2^ was computed as: $\mathrm R^2=1-\left({\textstyle\underset{}{\sum^{2}\left(\mathrm{full}\right)}}\right)/{\textstyle\underset{}{\sum^{2}\left(\mathrm{null}\right)}}$; Where ∑^2^(full) is the residual variance from the TE-specific model and ∑^2^(null) is the residual variance from a null model that included only the intercept and the same phylogenetic correlation structure.

We also used a PGLS to test whether total GC content is associated with proportion of total TEs relative to the genome and genome size. To do this, we extracted the proportion of GC content and proportion of TEs from RepeatMasker summary table output. We then used the same PGLS method as above to test whether genome size and proportion of all TEs relative to the genome are associated with proportion of GC content. In addition, we investigated if the proportion of families of TEs are associated with the proportion of GC content. In our model, we used the proportion of GC content as the response variable, with genome size and the proportion of TEs as the predictor. In addition, we controlled for contig N50 for each genome to isolate confounding factors by including it as a predictor variable in our model. Finally, we used a Benjamini-Hochberg procedure to control for False Discovery Rate.

Finally, we use PGLS to test whether the abundance of specific families of TE are associated with clutch size and larval period across anurans. Because several data points for larval period and clutch size were reported as ranges, we conducted three separate PGLS analyses: (1) the minimum value of the range, (2) the maximum value of the range, and (3) the mean of the range. We removed *Eleutherodactylus coqui* from the larval period analysis, as this species exhibits direct development. We could only find clutch size data for 55 species (Table S2) and larval period data for 39 species (Table S3). In our models, number of elements for each TE class, length occupied by each TE class, and proportion of TE relative to the entire genome were used as the response variable, with life history and ecological traits as the predictor. To isolate assembly contiguity as a confounding factor, we controlled for contig N50 for each genome by including it as a predictor variable in our models. Finally, we used a Benjamini-Hochberg procedure to control for False Discovery Rate [67].

### Phylogenetic signal and ancestral character state reconstruction

We examined phylogenetic niche conservatism (PNC) in genome size and TE content (percent TE’s, TE count, and TE length) using two complementary measures of phylogenetic signal: Blomberg’s *K* [68] and Pagel’s *λ* (Pagel, 1999) [69]. Blomberg’s *K* assesses whether closely related species resemble each other more or less than expected under a Brownian motion model of trait evolution. A *K* value of 1 indicates that trait variation closely matches the Brownian motion expectation. Values of *K* less than 1 suggest that traits are more evolutionarily labile than expected. Values greater than 1 indicate greater similarity among relatives than expected under Brownian motion. Pagel’s *λ* quantifies the extent to which phylogeny predicts trait variation across species and ranges from 0 to 1, with 0 indicating no phylogenetic signal (i.e., a star phylogeny where all species are equally related), and 1 indicating trait evolution consistent with Brownian motion given the tree’s topology and branch lengths [70].

We used the *‘phylosig’* function from the *phytools* R package [71] to estimate both K and λ, running 1,000 simulations to assess whether the observed values significantly differed from zero. To test the significance of phylogenetic signal, we compared the observed *K* values to those obtained through iterative randomizations in which tip labels were shuffled [72]. Finally, we modeled the evolutionary history of genome size and TE content using a maximum likelihood approach. We used the function *‘anc.ML’* in the *phytools* package [71] to conduct ancestral character state reconstructions of genome size, TE percent, TE length, and TE count. We calculated 95% confidence intervals (CIs) for the estimated ancestral node values, and assessed overlap of the CIs between adjacent nodes. Non-overlapping 95% CIs between parent and child nodes were considered significant evolutionary transitions. We calculated Pearson’s correlation coefficient to evaluate whether these TE metrics were positively associated with genome size using the *‘cor.test’* function (method = “pearson”) in the *stats* R package [73].

### Genome size and repeat landscape skewness

We used the RepeatMasker alignment output file and the RepeatMasker utility script calcDivergenceFromAlign.pl to estimate repeat landscapes for each anuran genome. The calcDivergenceFromAlign.pl utility script calculates the percent divergence of each repeat element identified in the genome from the element consensus sequence in the repeat library supplied to RepeatMasker using the Kimura 2-parameter divergence metric (K2P) [74]. K2P accounts for nucleotide substitution rate differences between transitions and transversions, and can be used as a proxy for TE age, with higher divergence indicating older elements that have accumulated more mutations over time. Per-element divergence estimates were placed into 1% bins that ranged from 0 to 50%. We focused on the classes of known transposable elements: DNA transposons, LINEs, LTR retrotransposons, and SINEs. We then calculated repeat landscape distribution skewness for each anuran genome using the R package moments [75]. A right skewed distribution indicated a shift towards less divergence from repeat library consensus sequences and a more active TE landscape. A negatively skewed distribution indicated a shift towards more divergence from repeat library consensus sequences, and a less active transposable element landscape. Skewness was then regressed against log_10_ genome size using the “brms” R package [76].

### Genome size and active Ty3 elements

Based on the finding that the abundances of TEs belonging to the Ty3, L1, Penelope, and Copia families were associated with genome size across anurans (see Results), we evaluated the relationship between the proportion of each genome occupied by recently active insertions from these families and genome size. We used the output from calcDivergenceFromAlign.pl to evaluate the relationship between log_10_ genome size and the proportion of the genome composed of elements with ≤ 10% divergence from the RepeatModeler consensus sequence. Based on visual inspection of a time scaled phylogeny of anuran families generated from the TimeTree database [77] (Figure S1), we determined that almost all major extant anuran families had diverged by ~ 30 million years ago. We then used published anuran substitution rates (1.0–3.0 × 10^− 9^ substitutions/site/year [78] to estimate that 10% K2P divergence from consensus represents 100 to 30 million years. Therefore, we considered TE insertions with ≤ 10% K2P to be recently active.

Given the lack of a relationship between recently active L1 and Penelope elements, and a weak relationship between Copia elements and genome size across anurans (see Results), we focused subsequent analyses on Ty3 elements. We used the RepeatMasker.out file to extract Ty3 elements that were ≥ 90% of the RepeatModeler consensus sequence and ≥ 4,000 bp, which is the minimum length of a fully intact Ty3 element [79]. Next, we identified open reading frames (ORFs) using NCBI ORFfinder [80], and classified ORFs as either retroviral protease, integrase, reverse transcriptase or Pol polyprotein with diamond blastp [81] and the NCBI protein database. Blastp hits were considered significant at e-value ≤ 1.0 × 10^− 24^. The per genome count of ORFs identified as being part of the Pol polyprotein were regressed against log_10_ genome size with a negative binomial link function using R package brms [82]. In both analyses relating genome size to active Ty3 elements we controlled for phylogenetic relatedness using an anuran species tree covariance matrix generated using R package “ape” [64] function vcv.phylo. We assessed model fit using a Bayesian *R*^2^ estimate using the function “bayes_R2” in R package “brms”. All Bayesian analyses were run using four chains, with 10,000 iterations. We observed chain convergence for all parameters in our analyses with R-hat values of 1.0.

## Results

### De novo repeat annotation

**Fig. 2:**
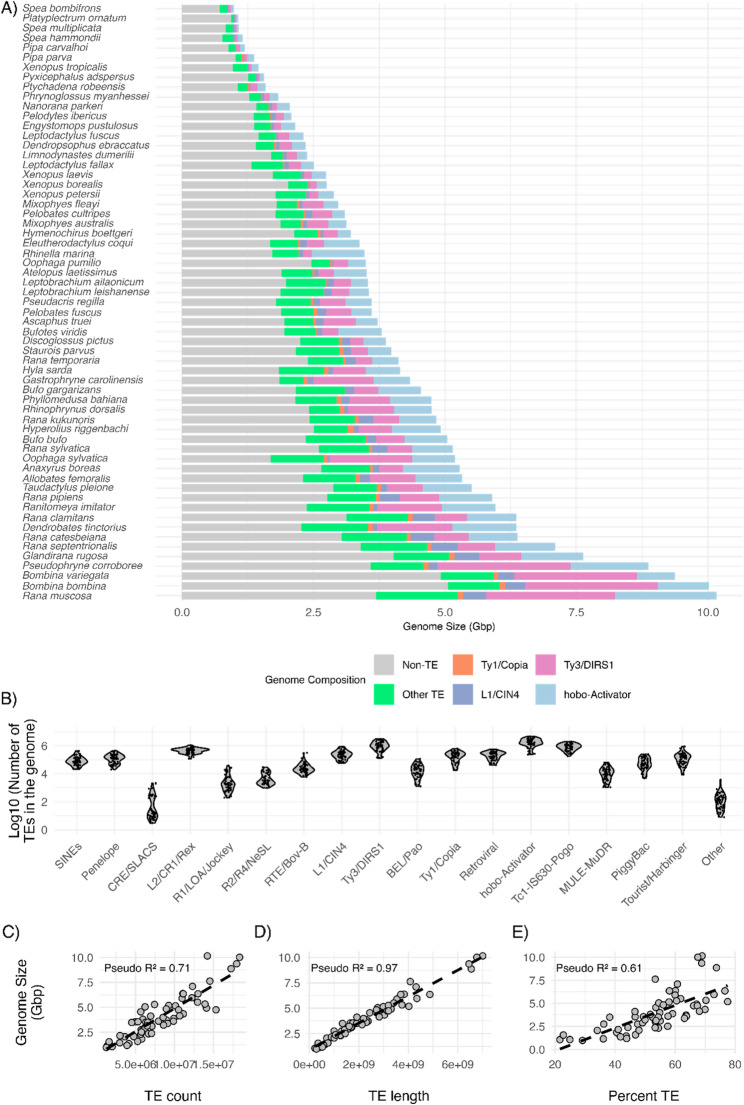
**(A)** Genome composition across species showing the relative contribution of different transposable elements (TE) families and non-TE regions. Bars represent total genome size partitioned into Non-TE sequences (grey), individual TE families (Ty1/Copia, L1/CIN4, Ty3/DIRS1, hobo-Activator), and grouped other TE elements. Species are ordered by decreasing genome size along the y-axis. **(B)** Distribution of TE insertions across major families of TE families. Violin plots show the distribution of log10-transformed TE insertion numbers for individual TE families across sampled genomes. Each point represents the number of insertions from a given TE family within a single genome. **(C)** Scatterplot showing the relationship between genome size (Gbp) and total TE insertions. **(D)** Relationship between genome size (Gbp) and total TE length. E Relationship between genome size (Gbp) and total proportion of the genome that are TEs. In C-E, each point represents a species, and dashed lines indicate linear regeneration fits. Pseudo *R*^2^ values are derived from simplified generalized partial least square models fitted to each dataset

We examined the repeat landscape among publicly available anuran genomes to examine patterns of TE abundance and genome size and found a large range in both genome size and TE proportion (Fig. 2A and B). We found that the *Platyplectrum ornatum* genome (Family: Myobatrachidae), which is 1.07 Gbp in size, had the lowest proportion of TEs compared to all other analyzed anuran genomes, with 23.97% (retroelements: 7.21%; DNA transposons: 14.39%), and that the *Rana mucosa* genome (Family: Ranidae), which is 10.1 Gbp in size, had the highest proportion of TEs, with 85.08% (retroelements: 36.42%; DNA transposons: 32.56%). We found that the genome with the lowest proportions of retroelements was *P. ornatum* (7.59%, genome size: 1.07 Gbp), while the genome with the highest proportion of retroelements was *Oophaga sylvatica* (Family: Dendrobatidae, 43.09%, genome size: 5.19 Gbp). The average percent of the genome consisting of retroelements across anurans was 24.39%. We found that the lowest proportion of DNA transposons in an anuran genome was 5.25% in *P. ornatum* (genome size: 1.07 Gbp) while the highest proportion of DNA transposons was 36.69% (*Rhinella marina*, Family: Bufonidae, genome size: 3.47 Gbp). The average percent of the genome composed of DNA transposons across all analyzed anurans was 20.38%. Collated results of repeat landscapes across all 61 genomes can be found in Supplementary Table 1.

### Transposable elements correlate with genome size variation in anurans

We found a positive correlation between genome size and the overall number of TE insertions (estimate = 5.040E-07, q-value = 8.880E-21, pseudo-*R*^2^ = 0.97) (Fig. 2C), the overall length of TE insertions (estimate = 1.259E-09, q-value = 1.130E-41, pseudo-*R*^2^ = 0.71) (Fig. 2D), and the overall proportion of TE relative to the entire genome (estimate = 0.105, q-value =,1.216E-09 pseudo-*R*^2^ = 0.614) (Fig. 2E). We found a significant correlation between size of a genome and the number of inserts belonging to three retroelements, L1/CIN4 (estimate = 2583.007, q-value = 0.003708, pseudo-*R*^2^ = 0.7), Ty1/Copia (estimate = −5504.96, q-value = 0.002479, pseudo-*R*^2^ = 0.33), Ty3/DIRS1 (estimate = 1567.501, q-value = 4.64E-05, pseudo-*R*^2^ = 0.74), and one DNA transposon, hobo-Activator (estimate = 769.2931, q-value = 0.021449, pseudo-*R*^2^ = 0.84) (Table 2). Slope coefficients for the PGLS analysis indicate that L1/CIN4, Ty3/DIRS1, and hobo-Activator TE families increased with genome size, while Ty1/Copia TE family decreased with genome size. The λ value for this model was − 0.08. Note that although we report λ for models here and below, this value generally should not be interpreted as a phylogenetic signal because in PGLS models it incorporates extra variation including model residuals [83]. We found a significant correlation between size of a genome and the total length of inserts belonging to four retroelements, Penelope (estimate = 13.39766, q-value = 9.8E-05, pseudo-*R*^2^ = 0.36), L1/CIN4 (estimate = 3.822462, q-value = 5.62E-05, pseudo-*R*^2^ = 0.6), Ty1/Copia (estimate = 12.97867, q-value = 0.000733, pseudo-*R*^2^ = 0.58), and Ty3/DIRS1 (estimate = 1.569828, q-value = 1.53E-10, pseudo-*R*^2^ = 0.81) (Table 3). Slope coefficients for the PGLS analysis indicate that all significant TE families increased with genome size. The λ value for this model was 0.32. We did not find a significant correlation between size of a genome and the proportion of inserts relative to the entire genome (Table 4). The λ value for this model was 1.02.

**Table 2:** Summary of PGLS test when using genome size as the response variable and the number of elements for each TE class as the predictor variable. Bold represents statistically significant results (q-value < 0.05)

Lambda		TE Class		Estimate	Std Error	t value	*p*-value	q-value
−0.08		(Intercept)		2.98E + 08	1.74E + 08	1.706695	0.095443	0.318144
R E T R O E L E M E N T S		SINEs		597.432	1483.159	0.402811	0.68918	0.8108
		Penelope		2429.004	1565.837	1.55125	0.128528	0.367222
		L I N E s	CRE/SLACS	89549.43	189602.8	0.4723	0.639217	0.8108
			L2/CR1/Rex	182.0459	449.6726	0.404841	0.687699	0.8108
			R1/LOA/Jockey	−5275.7	11097.91	−0.47538	0.63704	0.8108
			R2/R4/NeSL	1626.866	17708.59	0.091869	0.92725	0.92725
			RTE/Bov-B	3573.898	1654.465	2.160154	0.036658	0.146633
			**L1/CIN4**	**2583.007**	**689.8279**	**3.744423**	**0.000556**	**0.003708**
		L T R	BEL/Pao	796.0839	4798.304	0.165909	0.869044	0.92725
			**Ty1/Copia**	**−5504.96**	**1371.517**	**−4.01377**	**0.000248**	**0.002479**
			**Ty3/DIRS1**	**1567.501**	**285.7061**	**5.486409**	**2.32E-06**	**4.64E-05**
			Retroviral	−769.46	1261.072	−0.61016	0.545121	0.8108
D N A	T R A N S P O S O N S	**hobo-Activator**		**769.2931**	**254.3898**	**3.024072**	**0.00429**	**0.021449**
		Tc1-IS630-Pogo		167.1152	328.82	0.508227	0.614018	0.8108
		MULE-MuDR		11006.11	9526.914	1.155265	0.254671	0.636677
		PiggyBac		1379.461	1736.184	0.794536	0.431459	0.8108
		Tourist/Harbinger		−544.246	753.7819	−0.72202	0.47438	0.8108
		Other (Mirage, P-element, Transib)		−107409	168789.2	−0.63635	0.528085	0.8108
Contig N50				1.087425	7.420135	0.146551	0.884205	0.92725

**Table 3:** Summary of PGLS test when using genome size as the response variable and length occupied for each TE class as the predictor variable. Bold represents statistically significant results (q-value < 0.05)

Lambda		TE Class		Estimate	Std Error	t value	*p*-value	q-value
0.32		(Intercept)		0.955778	0.135428	7.057488	1.37E-08	1.37E-07
R E T R O E L E M E N T S		SINEs		−1E-09	2.21E-09	−0.46948	0.641214	0.723342
		**Penelope**		**1.34E-08**	**2.78E-09**	**4.825932**	**1.96E-05**	**9.8E-05**
		L I N E s	CRE/SLACS	−3.6E-07	4.39E-07	−0.81648	0.418943	0.679829
			L2/CR1/Rex	5.69E-10	9.57E-10	0.594522	0.55543	0.723342
			R1/LOA/Jockey	−4.5E-08	2.69E-08	−1.68292	0.099993	0.249982
			R2/R4/NeSL	2.04E-08	5.05E-08	0.403609	0.688598	0.72484
			RTE/Bov-B	3.26E-09	4.19E-09	0.776538	0.441889	0.679829
			**L1/CIN4**	**3.82E-09**	**7.51E-10**	**5.08844**	**8.43E-06**	**5.62E-05**
		L T R	BEL/Pao	9.91E-10	2.17E-09	0.455703	0.651008	0.723342
			**Ty1/Copia**	**1.3E-08**	**3.16E-09**	**4.112827**	**0.000183**	**0.000733**
			**Ty3/DIRS1**	**1.57E-09**	**1.66E-10**	**9.44376**	**7.66E-12**	**1.53E-10**
			Retroviral	−1E-09	1.27E-09	−0.82722	0.412904	0.679829
D N A	T R A N S P O S O N S	hobo-Activator		6.02E-10	3.87E-10	1.555649	0.127478	0.283284
		Tc1-IS630-Pogo		−5.8E-12	6.72E-10	−0.00857	0.993202	0.993202
		MULE-MuDR		4.32E-08	1.94E-08	2.226412	0.031541	0.105138
		PiggyBac		−4.1E-09	2.14E-09	−1.89713	0.06487	0.185343
		Tourist/Harbinger		6.56E-10	1.22E-09	0.540064	0.592074	0.723342
		Other (Mirage, P-element, Transib)		6.6E-07	8.35E-07	0.790204	0.433956	0.679829
Contig N50				−3.7E-09	5.48E-09	−0.67958	0.500587	0.715124

**Table 4:** Summary of PGLS test when using genome size as the response variable and percent for each TE class as the predictor variable.

Lambda		TE Class		Estimate	Std Error	T value	p -value	q-value
1.02		(Intercept)		1.172368	1.072637	1.092977	0.280784	0.395313
R E T R O E L E M E N T S		SINEs		0.848924	0.359192	2.363427	0.022929	0.123647
		Penelope		0.379198	0.35457	1.06946	0.291118	0.395313
		L I N E s	CRE/SLACS	−82.1398	51.21214	−1.60391	0.11641	0.355989
			L2/CR1/Rex	0.121876	0.192942	0.631672	0.531107	0.590119
			R1/LOA/Jockey	−5.75939	4.391401	−1.31152	0.196981	0.395313
			R2/R4/NeSL	−1.14262	7.848489	−0.14559	0.884962	0.884962
			RTE/Bov-B	−1.53391	0.686248	−2.23522	0.030912	0.123647
			L1/CIN4	0.639272	0.235204	2.71795	0.009582	0.117309
		L T R	BEL/Pao	0.68383	0.296287	2.307997	0.02612	0.123647
			Ty1/Copia	0.273673	0.405105	0.67556	0.503113	0.590119
			Ty3/DIRS1	0.343953	0.130382	2.638042	0.011731	0.117309
			Retroviral	−0.16813	0.117708	−1.42839	0.160755	0.395313
D N A	T R A N S P O S O N S	hobo-Activator		0.061157	0.057833	1.057477	0.296485	0.395313
		Tc1-IS630-Pogo		−0.09427	0.082192	−1.14693	0.258061	0.395313
		MULE-MuDR		0.769332	2.44186	0.31506	0.754314	0.794015
		PiggyBac		−0.41971	0.267693	−1.56788	0.124596	0.355989
		Tourist/Harbinger		−0.20376	0.177922	−1.14521	0.258764	0.395313
		Other (Mirage, P-element, Transib)		168.6559	149.5963	1.127407	0.266125	0.395313
Contig N50				−1E-08	1.36E-08	−0.75645	0.453704	0.56713

### Ancestral character state reconstruction and phylogenetic signal

**Fig. 3:**
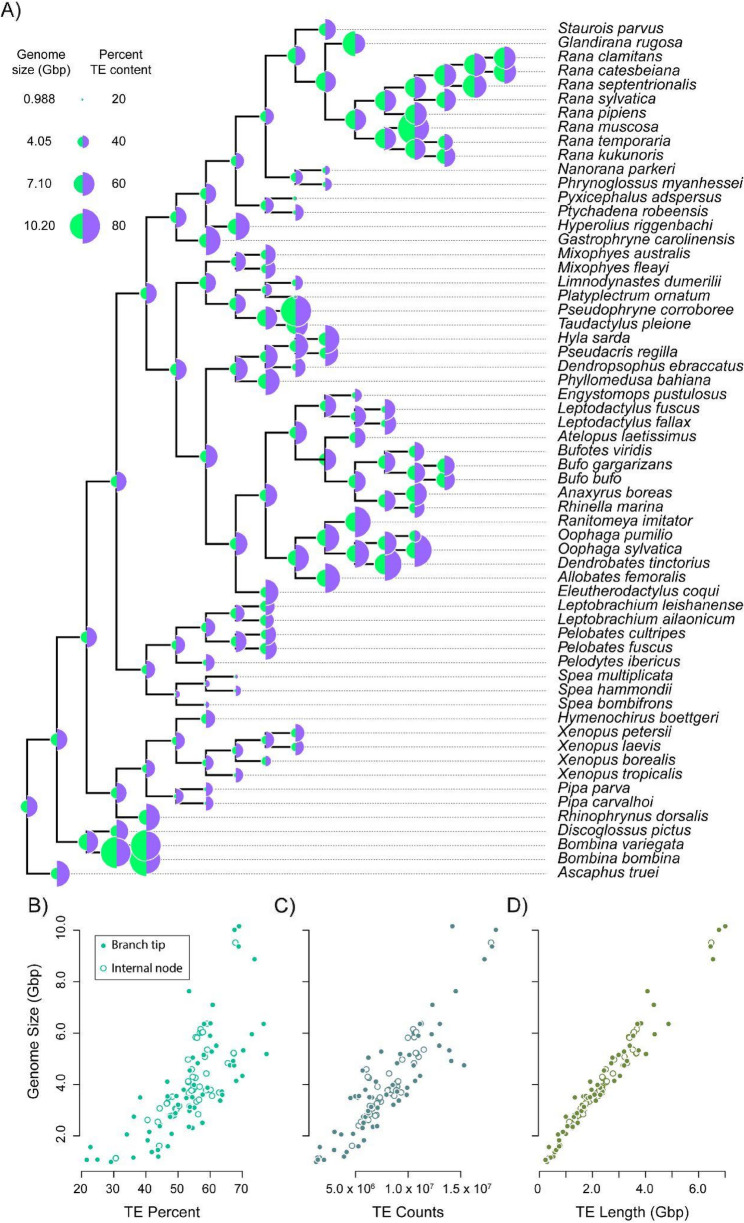
Ancestral character state reconstructions of genome size and TE content and associations between TE content and genome size. **(A)** A maximum likelihood approach for ancestral character state reconstruction revealed a general pattern of gradual evolution of genome size (green) and TE percent (purple). Point size at the branch tips and internal tree nodes are scaled to the range of values for each trait, and highlight concordance in the observed evolutionary patterns. We tested evolutionary correlations using Pearson’s correlation coefficient. All measures of TE content **(B)** TE percent, **(C)** TE count, and **(D)** TE length are significantly and positively related (all Pearson’s *r* > 0.70, all *p* < 0.001) to genome size (y-axis) across tree tips (closed points) and internal nodes of the tree (open points)

**Fig. 4:**
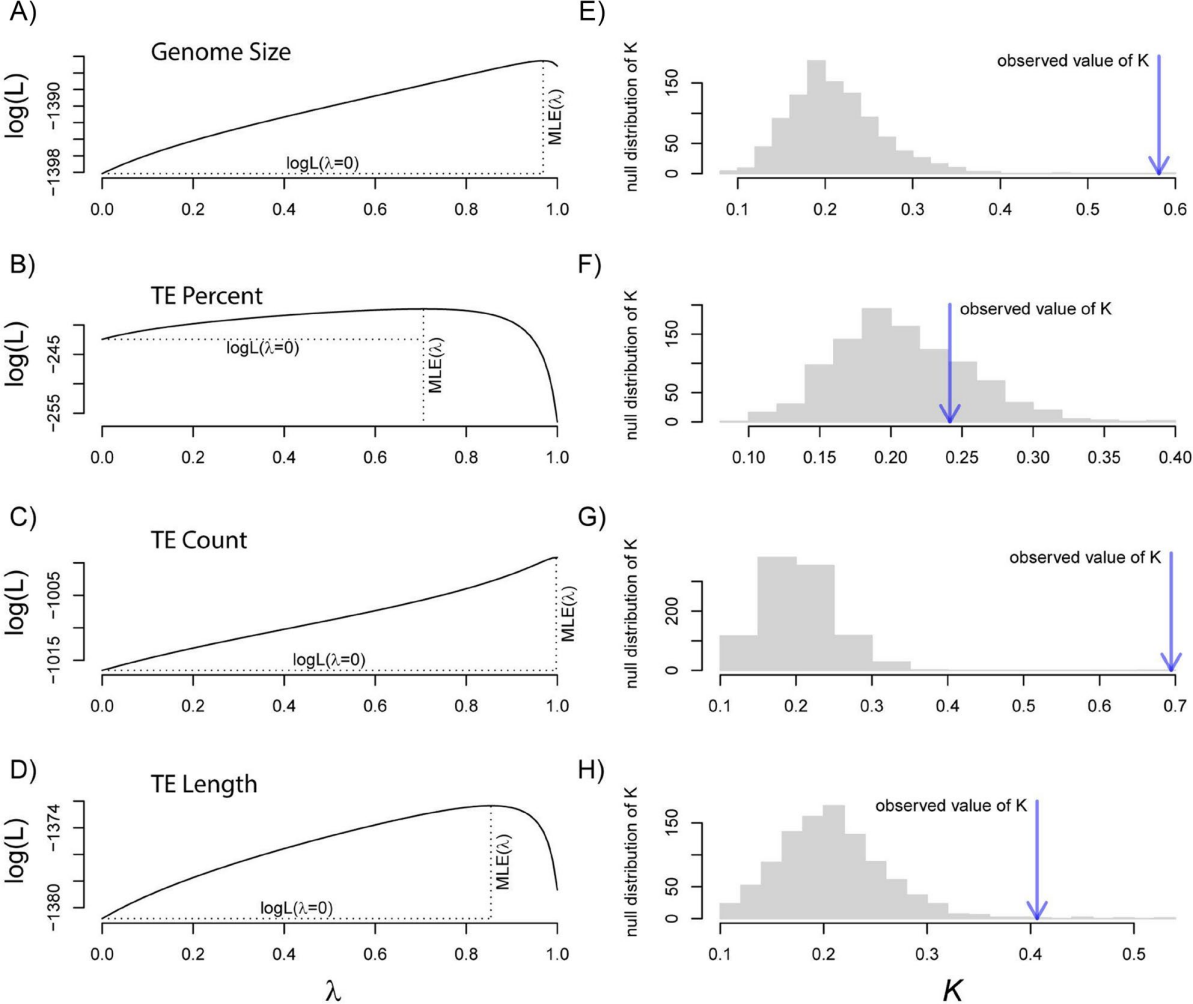
Phylogenetic signal of genome size and TE content. Likelihood surfaces for our estimates of Pagel’s 𝜆 for (**A**) genome size, (**B**) TE percent, (**C**) TE count, and (**D**) TE length. Values of 𝜆 approaching 1 show increasing phylogenetic influence. (**E - H**) Observed values of Blomberg’s *K* (blue arrows) superimposed over distributions of *K* estimated from 10,000 tip shuffling randomizations (gray histograms). Observed values to the right of the distribution medians signify relative evolutionary conservatism

Ancestral character state reconstruction showed strong patterns of correlated evolution between genome size and TE content. Genome size showed a general pattern of gradual evolution, with broadly overlapping confidence intervals (CIs) between all but one parent/child node pairings (see Fig. 3, Figure S2, and Table S4). The exception was in the branch leading to the genus *Bombina*. TE content (TE percent, TE count, TE length) similarly showed few significant evolutionary transitions, again with only a few exceptions (see Fig. 3, Supplementary Figure S2, and Supplementary Table S4). All measures of TE content were strongly and positively associated with genome size (all Pearson correlation coefficients *r* > 0.7, all *p* < 0.001; Fig. 3B, C and D). Together these results demonstrate a general pattern of gradual and correlated evolution in genome size and TE content among anurans. The gradual nature of genome and TE evolution in anurans is also reflected in our analyses of phylogenetic signal. Both Blomberg’s *K* and Pagel’s 𝜆 showed strong phylogenetic signal; 𝜆 values were near 1 for genome size and all measures of TE content (all 𝜆 >0.7; Fig. 4; Table 5). Blomberg’s *K* was more variable: *K* was 0.58 for genome size, 0.69 for TE count, 0.41 for TE length, and 0.24 for TE percent. The *K* estimate for TE percent was not statistically distinct from 0 (*p* = 0.23, Table 5). In all cases, the calculated values of *K* were greater than the median values produced by our tip-shuffling randomization (see Fig. 4), indicating that there is a strong phylogenetic signature of genome size and TE content that is more evolutionarily conserved than would be expected by random.

**Table 5:** Phylogenetic signal results. Pagel’s 𝜆 and blomberg’s *K* were estimated using the ‘*phylosig*’ function in the *phytools* R package. *P*-values test the signal metrics against a null of 0 (no signal)

	𝞴	*P*_𝞴=0_	K	*P*_K=0_
Genome Length	0.969	< 0.001	0.581	0.001
TE Percent	0.706	0.001	0.242	0.227
TE Count	0.997	< 0.001	0.694	0.001
TE Length	0.854	< 0.001	0.406	0.005

### Recently active Ty3 elements correlate with anuran genome size

**Fig. 5:**
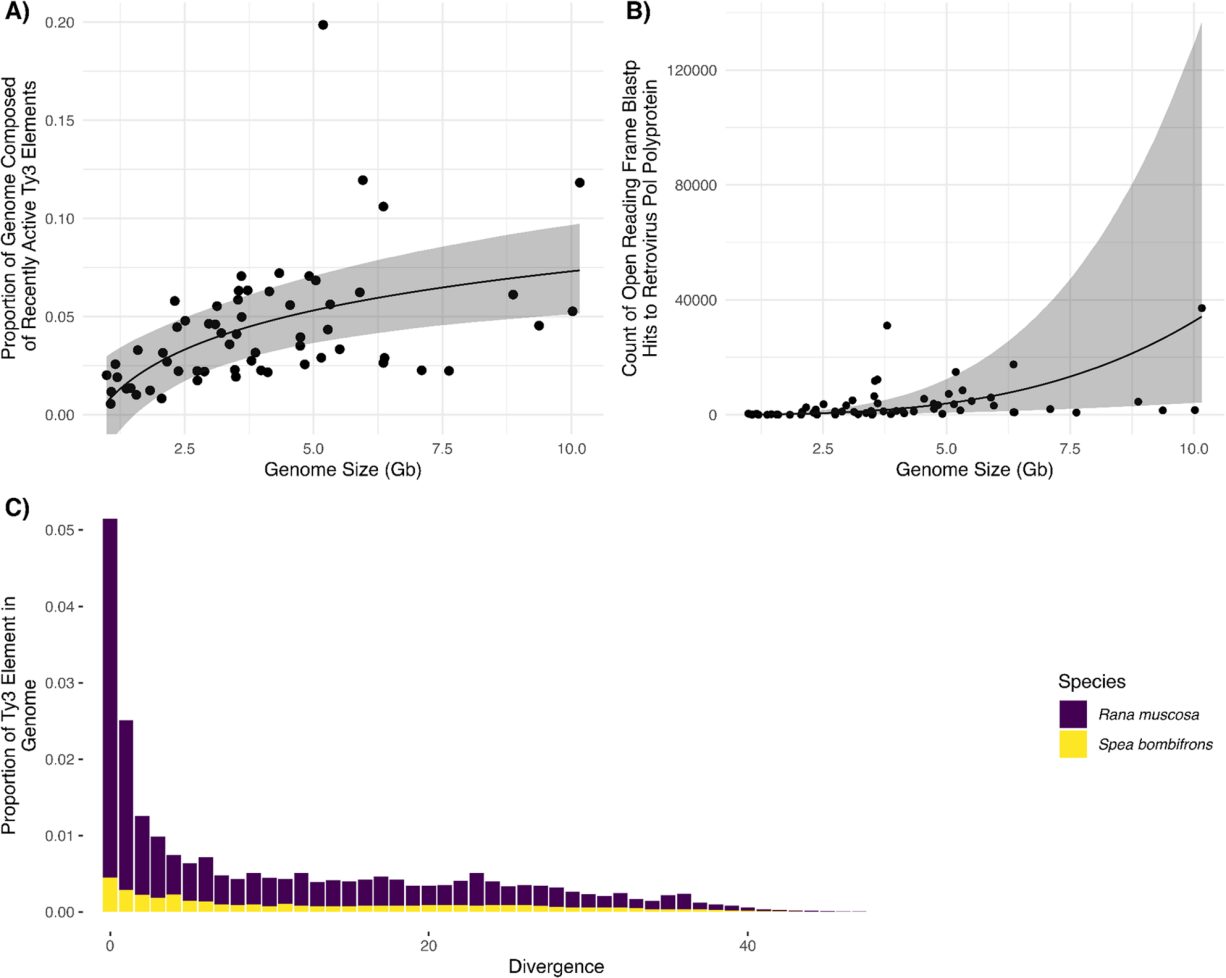
**(A) **Relationship between proportion of amphibian genome composed of recently active Ty3 elements and genome size. Elements were considered recently active if the integrated genome sequence had less than 10% divergence from Ty3 RepeatModeler consensus sequences. Each point represents the per species genome proportion composed of recently active Ty3 elements. Solid line shows the significant relationship from the fitted model (Table S5) with 95% Credible Interval. Estimated Bayesian *R*^2^ 0.38 (95% CI: 0.15, 0.57). Model was fit with genome size on log_10_ scale. **(B)** Relationship between the count of open reading frame (ORF) blastp hits to retroviral Pol polyprotein from putatively full length Ty3 elements and genome size where count of blastp hits follows a negative binomial distribution. Elements were considered full length if they were greater than 90% of the RepeatModeler consensus sequence and greater than 4,000 bp in length. Count includes ORFs that were identified as either integrase, protease, reverse transcriptase, or Pol polyprotein. ORFs were found using ORFfinder with full length TY3 elements as input. Each point represents the per species count of ORF blastp hits to retroviral Pol polyprotein. Solid line shows the significant relationship from the fitted model (Table S7) with 95% Credible Interval. Estimated Bayesian *R*^2^ 0.53 (95% CI: 0.27, 0.74). The model was fit with genome size on log_10_ scale. **(C)** Ty3 repeat landscape for the species with the smallest, *Spea bombifrons*, and largest, *Rana muscosa*, genome in our dataset. Divergence between the Ty3 element and RepeatModler consensus sequence was calculated using the RepeatMasker utility script calcDivergenceFromAlign.pl. Per-element divergence estimates were placed into 1% bins that ranged from 0–50%

We did not identify a significant relationship between repeat landscape skewness and genome size (β = 2.54; 95% CI −6.49–11.68%). Further, there was no significant relationship between genome size and recently active L1 (OLS regression *p*-value = 0.96) (Figure S3) and Penelope elements (OLS regression *p*-value = 0.37) (Figures S4), and a very weak, positive relationship between recently active Copia elements and genome size (OLS regression *p*-value = 0.014, *R*^2^ = 0.098; Figure S5). However, we found a strong positive relationship between both measures of active Ty3 elements and genome size. The proportion of the genome composed of recently active Ty3 elements was positively correlated with anuran genome size (β = 0.06; 95% CI 0.03–0.09; Fig. 5A; Table S5), with no effect of phylogeny (𝜆 = 0), and an estimated Bayesian *R*^2^ 0.38 (95% CI 0.16–0.56).

After filtering for full length Ty3 elements, we were able to identify Ty3 Pol polyprotein ORFs in every anuran species with the exception of *Phrynoglossus myanhessei* and *Platyplectrum ornatum*. Further, after excluding *Ph. myanhessei* and *Pl. ornatum*, the count of Ty3 Pol polyprotein ORFs ranged from 14 in *Oophaga pumilio* to 37,120 in the species with the largest genome, *Rana muscosa* (Table S6). We found a strong positive relationship between the count of Ty3 Pol polyprotein ORFs and anuran genome size (β = 6.55; 95% CI 4.12–9.11; Fig. 5B; Table S7), with a weak effect of phylogeny (𝜆 = 0.12; 95%CI 0.06–0.20), and an estimated Bayesian *R*^2^ 0.53 (95% CI 0.27–0.74). We compared results from our Bayesian approach to a PGLS analysis and the significant association between genome size and recently active Ty3 elements remained.

### Genome size and proportion of GC content

We found a significant correlation between proportion of GC content and proportion of total TEs relative to the genome (estimate = 0.055, q-value = 2.049E-20, pseudo-*R*^2^ = 0.414), however, we did not find a significant correlation between GC content and genome size (estimate = 0.169, q-value = 0.081, pseudo-*R*^2^ = 0.278) (Table S8). The slope coefficient for the PGLS analysis for genome size and proportion of interspersed repeats were positive, indicating that genome size and proportion of interspersed repeats increased with proportion of GC content. The λ value for this model was 1.02. In addition, we found an association between proportion of GC content and the number of insertions belonging to the TE family Ty3/DIRS1 (estimate = 2.254E-06, q-value = 0.042, pseudo-*R*^2^ = 0.338) (Table S9). Slope coefficients for the PGLS analysis indicate that insertions of Ty3/DIRS1 increased with proportion of GC content. We did not find an association between proportion of GC content and both length (Table S10) and proportion of TE relative to the genome (Table S11).

### Transposable elements and life history traits

We examined how TE content is related to life history traits that have been predicted to be related to TE content, clutch size and larval period. Both are variable in many species, so as a result we examined minimum and maximum for both clutch size and larval period, as well as average clutch size. We did not find a significant correlation between minimum, maximum, or average clutch size and TEs. We did find a significant correlation between minimum larval period and both the number (estimate = 0.001, q-value = 0.007, pseudo-*R*^2^ = 0.619, Table S12) and length of TE insertions (estimate = 2.871E-06, q-value = 0.001, pseudo-*R*^2^ = 0.301, Table S13) of the TE family L2/CR1/Rex. Slope coefficients for the PGLS analysis indicate that the number and length of L2/CR1/Rex insertions increased with larval period. As for maximum larval period, we found a significant correlation between larval period and several families of TEs. There was an association between maximum larval period and the number of TE insertions for the TE family hobo-Activator (estimate = −7.185E-04., q-value = 0.029, pseudo-*R*^2^ = −0.040) and PiggyBac (estimate = −0.003, q-value = 0.047, pseudo-*R*^2^ = −0.059) (Table S14) and a correlation between maximum larval period and the length of TE insertions for the TE family L2/CR1/Rex (estimate = 4.436E-06, q-value = 0.013, pseudo-*R*^2^ = 0.106) and SINEs (estimate = 9.910E-06, q-value = 0.006, pseudo-*R*^2^ = − 0.206) (Table S15). Slope coefficients for the PGLS analysis indicate that the number of TE insertions for the family hobo-Activator and PiggyBac decreased with larval period and the length of the TE insertions for the family L2/CR1/Rex and SINEs increased with larval period. The same results are observed when testing whether there is an association between mean larval period and both the number of TE insertions and the length of TE insertions (Table S16-S17).

## Discussion

The remarkable diversity in genome size among anurans highlights the dynamic nature of genome evolution within this group. Anurans have sparked particular interest due to their extreme variability in genome size and composition, often possessing unusually large genomes compared to other vertebrates [84, 85]. In this study, we aimed to test if TEs are driving the evolution of genome size in frogs and toads and to identify specific families of TEs driving genome variation in a phylogenetic context. We found that 1) genome size is driven by TE content and that there is a strong phylogenetic signal to both, 2) genome size is driven by specific TE families, some of which have experienced recent or ongoing activity, and 3) that TE content is associated with life history traits such as larval period.

### Genome size is driven by TE composition and there is a strong phylogenetic signal to both

Our evaluations of total TE content, total number of insertions (the net outcome of both insertion and deletion events, that is, the number of insertions minus the number of deletions), and total TE length (in bases) are strongly correlated with genome size. This tracks with other systems, including insects, mammals, and fish [86–88]. Using ancestral character state reconstructions and phylogenetic analyses, we found that both genome size and TE content are strongly associated with phylogeny, and thus are mostly evolutionarily constrained. This is consistent with relatively gradual evolution of both TEs and genome size over evolutionary time. However, we also observed a significant discordance between ancestry and extant species in the lineage leading to the genus *Bombina.* Both *Bombina bombina* and *B. variegata* have extremely large genomes for frogs (10.02 and 9.37 Gbp respectively), and this is at odds with other early-diverging anurans, suggesting that a burst of TE activity may have driven the evolution of large genomes in this group. Similarly, frogs in the genus *Phyllobates* (Family: Dendrobatidae) exhibit a rapidly expanding genome size with several species possessing genomes >10 Gbp in length, similar to that found in Bombinatoridae [48, 89]. Conversely, while many of the ranid frogs in our study have evolved similarly large genomes, they do not exhibit a concomitant burst of TE activity. Instead, they show a more gradual accumulation of TEs, as evidenced by the overlapping confidence bounds at the base of the ranid lineage in our ancestral character state analyses. Collectively these results demonstrate that TE proliferation is a key mechanism driving anuran genome size evolution, and that it can proceed either gradually, as in the Ranidae, or spasmodically, as in the Dendrobatidae. As more data become available we will be better able to ascertain how frequently episodic bursts of TE content lead to dramatic changes in genome size.

### Transposable element families associated with genome size and recent retrotransposon activity

In addition to finding a general relationship between TE content and genome size, we also identified that four families of retroelements (Penelope, L1/CIN4, Ty1/Copia, and Ty3/DIRS) and one family of DNA transposon (hobo-Activator) are associated with increased genome size in anurans. These results suggest that the proliferation of TEs plays a key role in genome expansion in this lineage. Genome size showed a positive correlation with both the number and total length of L1/CIN4 and Ty3/DIRS1 insertions. However, genome size was negatively correlated with the number of Ty1/Copia elements while being positively correlated with the total length they occupy. We interpret this to mean that large genomes are more likely to possess intact Ty1/Copia, whereas species with smaller genomes are more likely to have truncated or degenerated Ty1/Copia sequences. Thus, although there may be many genomic Ty1/Copia regions, they are each short and thus have a small total length of Ty1/Copia regions in the genome. In the plant genus *Eleocharis*, larger genomes were associated with recent amplification and a greater proportion of intact Ty1/Copia elements than *Eleocharis* with smaller genomes [90]. In the case of Penelope, only the length of insertions was positively associated with genome size, suggesting that any expansion of genome size driven by these retroelements is primarily due to the insertion of relatively long sequences.

Numerous studies across the tree of life have shown that the proliferation of TEs is a primary driver of genome size variation [52, 91–94]. However, the relative contribution of TE insertion counts versus total DNA accumulated from TE is complex and remains an open question. For instance, the genome expansion in mammals is more closely associated with the total DNA gained through retrotransposon activity than with the frequency of insertion events [26]. Another study found that the wild rice *Oryza australiensis* underwent bursts of LTR retrotransposons elements, resulting in greater genome size increases than would be expected from gradual increases in insertion frequency alone [51]. These patterns align with our results in anurans, where the total length of the Penelope TE family was significantly associated with genome size, whereas the number of Penelope insertions was not. Interestingly, we also observed the opposite pattern for the hobo-Activator TE family, where genome size was correlated with the number of insertions but not with their total length. These results suggest this DNA transposon contributes to genome expansion through frequent transposition events and a higher number of smaller insertions. While some TE families show a stronger association with either length or copy numbers, increasing evidence suggests that both factors can act synergistically to drive genome expansion in amphibians. For instance, salamanders are known for their exceptionally large genomes, which have been attributed to both TE abundance and the accumulation of long, highly repetitive elements [95].

LTR retrotransposons such as the Ty1/Copia and Ty3/DIRS1 families, which were associated with genome size in our study, are a group of TEs often implicated in genome expansion in several systems [41, 52, 90, 96, 97]. When replicated, LTR elements can integrate sequences of DNA spanning several kilobases into the genome, some reaching up to 30 kilobases and larger [98]. Their ability to proliferate in bursts and accumulate large amounts of DNA makes them particularly effective at rapidly increasing genome size. This pattern aligns with other taxa such as plants and salamanders, where large genome sizes were found to be driven by the accumulation of LTR retrotransposons [41, 51].

To understand the relationship between TE activity and genome size we examined if recent activity of TE families that we found to be associated with genome size (L1, Penelope, Copia and Ty3) is a driving factor of genome size. To do this, we quantified the overall activity of the anuran repeat landscape using skewness (i.e., right skewed distribution indicating a more active TE landscape and negative skewed distribution indicating less active transposable element landscape), and evaluated the relationship between recently active components of repeat families L1, Penelope, Copia and Ty3 and genome size. While there was no correlation between genome size and overall repeat landscape activity, recently active L1, or recently active Penelope families, there were significant relationships between genome size and recently active Copia and Ty3 families. These results suggest that the activity of specific TE families, rather than overall TE activity, is contributing to genome size variation in anurans. While recently active Copia elements are associated with genome size, in our models the effect of Copia on genome size is minimal. However, we did find a strong relationship between recently active Ty3 elements and genome size. The importance of Ty3 elements to amphibian genomes has been documented [42], and likely exhibits species specific dynamics with expansions and contractions over evolutionary time [99]. This is consistent with our results showing weak to no phylogenetic signal on the relationship between recently active Ty3 elements and genome size. Further, we were able to identify Ty3 retroviral Pol polyprotein ORFs, including protease, integrase and reverse transcriptase, and correlate ORF abundance to genome size. The significance of this relationship indicates the machinery for active retrotransposition can be found in full length Ty3 elements and is associated with larger genome size. In sum, we found that not only is genome size associated with specific classes of TEs, but that *recent* activity of Ty3 is a predictor of genome size and that it shows species-specific patterns.

Our results identified that both genome size and proportion of interspersed repeats are significantly associated with proportion of guanine–cytosine (GC) content across anuran genomes. These findings suggest that nucleotide composition may be shaped by the expansion of repetitive elements, which is a pattern that has been observed in other vertebrate lineages [100]. Although genome size in our dataset is driven by TE content, most TEs, including the abundant retrotransposons in anurans, tend to be adenine–thymine (AT) rich [101]. For instance, L1 elements are universally AT-rich, but the proportion of AT pairs is especially high in mammals, lizards and frogs [102]. The chemical properties of GC base pairs enable them to influence the genome in several ways. For instance, GC pairs exhibit greater stability than AT pairs due to the presence of three hydrogen bonds, compared to the two formed by AT pair form. This additional hydrogen bond confers increased thermal stability and resistance to denaturation in GC–rich regions of DNA [103]. One hypothesis is that in larger genomes higher GC proportions are favored for stability, and as TEs get inactivated they are more likely to mutate over time to be more GC rich. Regardless, there is a clear relationship between genome size, TE content, and GC content which may be worth future exploration.

### Genome size is strongly constrained by life history traits

We found that two families of retroelements (L2/CR1/Rex and SINEs) and two families of DNA transposon (hobo-Activator and PiggyBac) are associated with larval development time. These results suggest that development duration may act as a major constraint on genome size, potentially influencing the retention or purging of TEs. Specifically, we found that the count and length of TEs in the L2/CR1/Rex family increased as the minimum larval development period increased. In contrast, as the maximum larval development period increased, the number of insertions from hobo-Activator and PiggyBac family decreased, while length of insertions from L2/CR1/Rex and SINEs family increased. These findings imply that different TE families may be subject to distinct selective pressures depending on developmental timing, reinforcing the idea that genome size evolution is shaped by life-history constraints. Retrotransposons such as L2/CR1/Rex and SINEs may influence development time through their impact on genome size and gene regulation. For instance, the TEs in the L2/CR1/Rex family are known to be typically long and have been shown to significantly contribute to genome size increases [104]. Studies suggest that genome size can influence cell size and replication, and as a result, species with larger genomes experience prolonged development periods [55, 105–107]. Development constraints, such as pressure for rapid development, could influence not only the rate of TE insertion but also the efficiency of TE removal. For example, species with shorter larval periods may be under stronger selection to maintain compact genomes and could therefore remove TEs more efficiently. These deletion dynamics may help explain why some TE families, such as hobo-Activator and PiggyBac, show decreasing copy numbers with increased development time, while others, like L2/CR1/Rex, increase in length. It is possible that the increased number and length of TE insertions play a pivotal role in the larval development of anurans and that species which need to replicate cells or develop faster than the norm need to more efficiently remove TE content from their genome.

In addition, beyond simply affecting the duration of development, genome size may also influence developmental complexity, such as the amount of morphological change that occurs during a given period. For example, amphibians display a wide range of developmental modes (e.g., biphasic metamorphosis and direct development), each involving different degrees of cellular and structural remodeling [108]. These differences in complexity may place distinct constraints on genome size, further shaping patterns of TE accumulation or removal. It is therefore possible that the increased number and length of TE insertions play a pivotal role not only in the duration but also in the complexity of larval development in anurans, and that species under pressure to develop quickly or with reduced complexity may need to more effectively remove TE content from their genome.

## Conclusion

Frogs and toads are a charismatic and widely distributed taxon with critical importance to ecosystems, which exhibits a remarkable diversity of genome sizes. In order to understand the patterns and processes driving genome size evolution in anurans, we annotated TEs in publicly available genome assemblies from 61 species representing 22 anuran families and tested models that predict the effects of TE diversity, abundance, and activity on genome size variation across the group. We confirm that TE abundance has a strong effect on genome size in anurans, there is a strong phylogenetic signature of genome size, and that TE content is more evolutionarily conserved than would be expected by chance. Further, we demonstrate that relatively recent activity of LTR retrotransposons, in particular the Ty3/DIRS1 family, has a strong effect on genome size variation across anurans. We also demonstrate that the total length and copy numbers of several types of TE, including retroelements and DNA transposons, are associated with larval period and developmental time across anurans, providing evidence that developmental processes place constraints on genome size and structure in amphibians.

## Supplementary Information

Supplementary Material 1.

Supplementary Material 2.

## Data Availability

All raw data for this manuscript were publicly available genome assemblies and downloaded from NCBI. The accession numbers for genomes used in this manuscript are listed in Table 1. All code used for analyses are available at 10.5281/zenodo.17074384.
