# Supplementary material for: Anuran genome size evolution is driven by relatively recent retrotransposon activity and by life history
Source: BMC Genomics. 2025 Dec 18;27:74. doi: 10.1186/s12864-025-12414-y (PMC12822217; doi:10.1186/s12864-025-12414-y)
Supplement: Supplementary file 2 — Supplementary Material 2. [file 12864_2025_12414_MOESM2_ESM.pdf]

Supplementary Figures

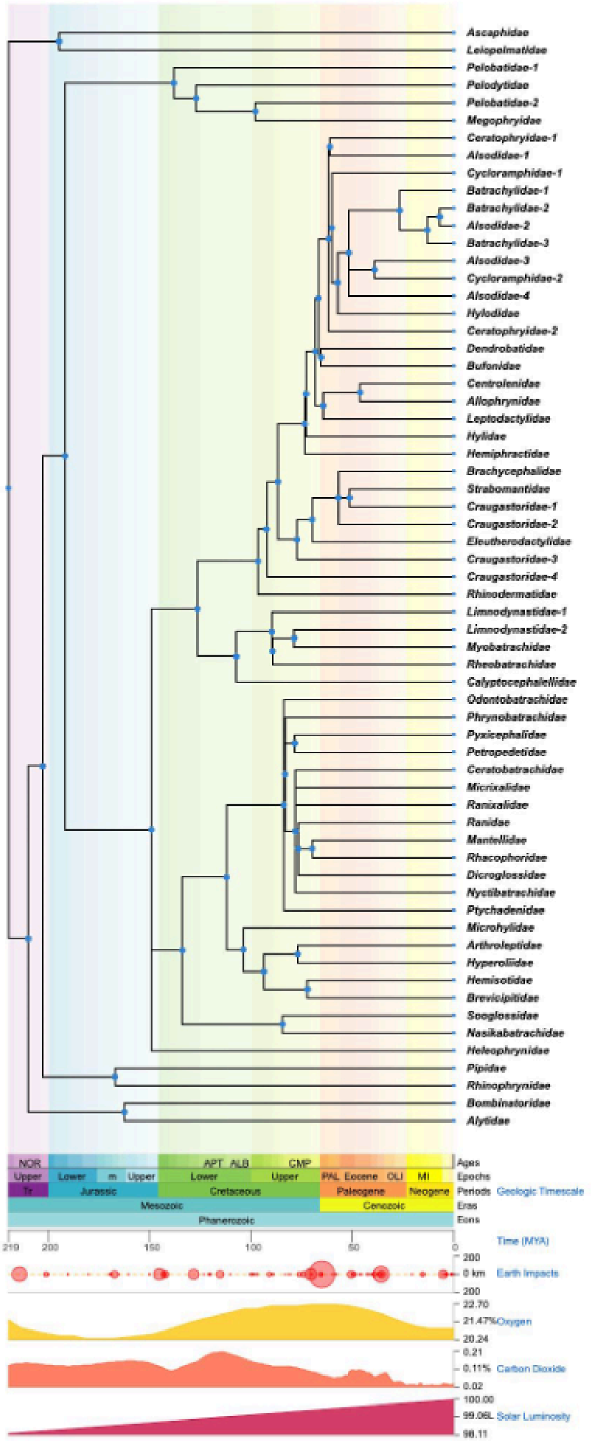

Figure S1: Time calibrated anuran family tree generated from Timetree.org.

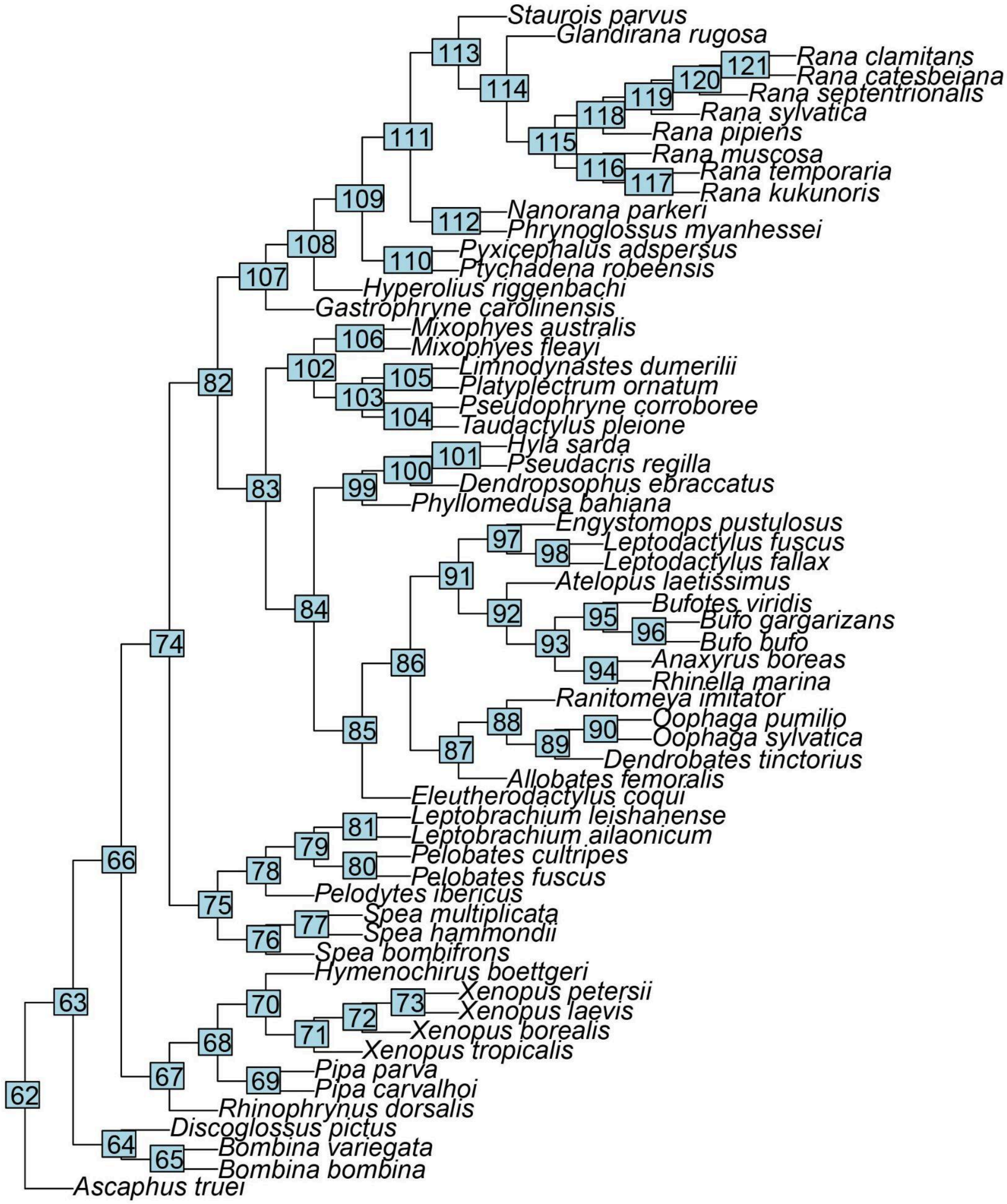

Figure S2. Anuran phylogeny with labeled nodes for reference in comparing overlapping confidence intervals for ancestral character state estimates (see Supplementary Table XX). All branch lengths set to 1 to highlight tree branching.

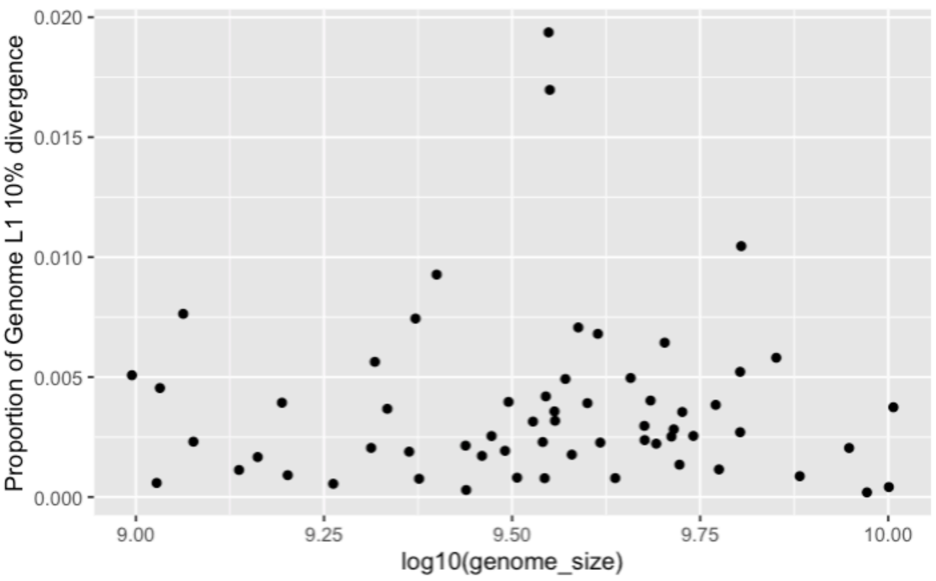

Figure S3: Relationship between proportion of amphibian genome composed of recently active L1 elements and genome size. Elements were considered recently active if the integrated genome sequence had less than 10% divergence from L1 RepeatModeler consensus sequences. Each point represents the per species genome proportion composed of recently active L1 elements (OLS regression p-value=0.95).

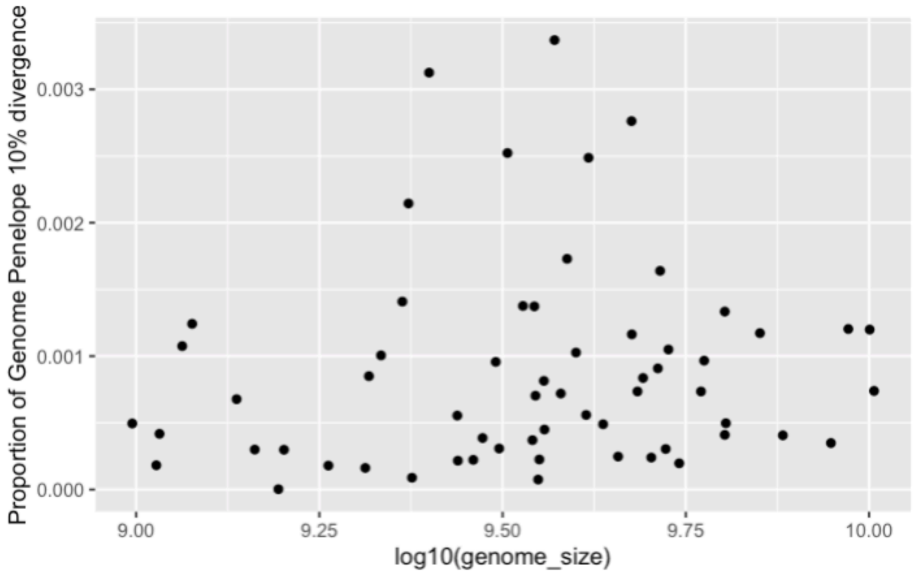

Figure S4: Relationship between proportion of amphibian genome composed of recently active Penelope elements and genome size. Elements were considered recently active if the integrated genome sequence had less than 10% divergence from Penelope RepeatModeler consensus sequences. Each point represents the per species genome proportion composed of recently active Penelope elements (OLS regression p-value = 0.37).

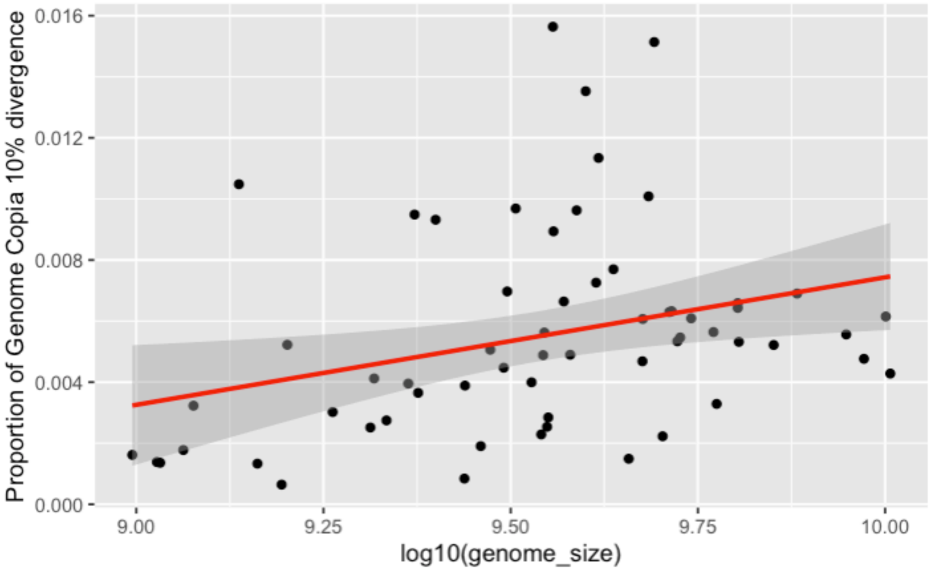

Figure S5: Relationship between proportion of amphibian genome composed of recently active Copia elements and genome size. Elements were considered recently active if the integrated genome sequence had less than 10% divergence from Copia RepeatModeler consensus sequences. Each point represents the per species genome proportion composed of recently active Copia elements (OLS regression p-value = 0.01, R2=0.09, β=0.004).
